# Dynamics of C-reactive protein and white blood cell count in critically ill patients with nosocomial Gram positive vs. Gram negative bacteremia: a historical cohort study

**DOI:** 10.1186/1471-2334-7-106

**Published:** 2007-09-14

**Authors:** Dominique M Vandijck, Eric A Hoste, Stijn I Blot, Pieter O Depuydt, Renaat A Peleman, Johan M Decruyenaere

**Affiliations:** 1 Department of Intensive Care Medicine, Ghent University Hospital – Ghent University, Faculty of Medicine and Health Sciences, De Pintelaan 185, Ghent, Belgium; 2 Department of Infectious Diseases, Ghent University Hospital – Ghent University, Faculty of Medicine and Health Sciences, De Pintelaan 185, Ghent, Belgium; 3 University College Ghent, Department of Health Care "Vesalius", Keramiekstraat 80, Ghent, Belgium

## Abstract

**Background:**

Nosocomial bacteremia is associated with a poor prognosis. Early adequate therapy has been shown to improve outcome. Consequently, rapid detection of a beginning sepsis is therefore of the utmost importance. This historical cohort study was designed to evaluate if different patterns can be observed in either C-reactive protein (CRP) and white blood cell count (WCC) between Gram positive bacteremia (GPB) vs. Gram negative bacteremia (GNB), and to assess the potential benefit of serial measurements of both biomarkers in terms of early antimicrobial therapy initiation.

**Methods:**

A historical study (2003–2004) was conducted, including all adult intensive care unit patients with a nosocomial bacteremia. CRP and WCC count measurements were recorded daily from two days prior (d_-2_) until one day after onset of bacteremia (d_+1_). Delta (Δ) CRP and Δ WCC levels from the level at d_-2 _onward were calculated.

**Results:**

CRP levels and WCC counts were substantially higher in patients with GNB. Logistic regression analysis demonstrated that GNB and Acute Physiology and Chronic Health Evaluation (APACHE) II score were independently associated with a CRP increase of 5 mg/dL from d_-2 _to d_+1_, and both were also independently associated with an increase of WCC levels from d_-2 _to d_+1 _of 5,000 × 10^3 ^cells/mm^3^.

**Conclusion:**

Increased levels of CRP and WCC are suggestive for GNB, while almost unchanged CRP and WCC levels are observed in patients with GPB. However, despite the different patterns observed, antimicrobial treatment as such cannot be guided based on both biomarkers.

## Background

Nosocomial bloodstream infection (BSI) is a major complication of intensive care unit (ICU) admission [1]. Physiological features such as fever, tachycardia and tachypnea have been proposed as indicators of sepsis [2,3]. These findings may be sensitive, but are less specific in the diagnosis of systemic inflammation or infection [4,5]. In this setting, C-reactive protein (CRP) and white blood cell counts (WCC) have been shown to be more reliable markers [6-8]. Early initiation of appropriate antimicrobials is a key to improve patients' survival [9]. Identification of the isolated pathogen including antibiogram is available at least 24-hrs after samples for blood cultures were performed. Early recognition of even the first minor signs of infection in case of a beginning bacteremia could therefore help to identify those patients who are more likely infected with either Gram positive or Gram negative pathogens [3]. The primary aim of our study was to investigate whether or not critically ill patients with nosocomial bacteremia caused by either Gram positive or Gram negative bacteria, present different patterns in the evolution of both biomarkers in order to facilitate decisions concerning the initial choice of an empiric antibiotic regimen.

## Methods

### Setting

The study was conducted in the Ghent University Hospital, a 1062-bed tertiary teaching care centre in Belgium. About 4100 patients are admitted to the 54-beds ICU annually.

### Study design

A historical observational cohort study of prospectively collected data (2003–2004) was performed in which all episodes of microbiological documented nosocomial bacteremia occurring in adult ICU patients (n = 174) were included. All data (i.e. demographic, clinical, laboratory, and physiological) were gathered by reviewing the charts and the computerized hospital laboratory and administrative databases.

Serial measurements of CRP and WCC serum concentrations were gathered, starting two days prior to onset of bacteremia (d_-2_) onwards until one calendar day (which is 48-hrs) after onset of bacteremia (d_+1_) to record the respective patterns of both biomarkers. To evaluate the evolution over time, delta (Δ) CRP levels and Δ WCC levels were calculated relative to the level at d_-2_. Patient characteristics, laboratory variables, and antimicrobial therapies were compared between episodes of Gram positive and Gram negative bacteremia. For analysis, only the first episode of BSI was considered.

### Definitions

Bacteremia was considered nosocomial when diagnosed ≥ 48-hrs upon initial hospitalization. Onset of bacteremia was defined as day 0 (d_0_), which corresponds to the day the first positive blood culture was sampled. In case of coagulase-negative Staphylococci, two positive blood cultures yielding coagulase-negative *Staphylococcus *on separate occasions within a 48-hrs period, and confirmation of clinical significance of bacteremia by the attending intensivist were required for diagnosis of bacteremia [10]. Blood cultures were routinely performed when the patients' temperature was ≥38.5°C or when infection was suspected on clinical grounds. Antimicrobial resistance and susceptibility was determined according to the guidelines as recommended by the National Committee for Clinical Laboratory Standards. Antibiotic therapy was defined as 'adequate' when the drug administered had in-vitro and clinical activity against the isolated strain and when initiated within 48-hours after sampling the positive blood culture. Therapy was considered 'inadequate' when there was no activity both, in-vitro and clinical against the isolated strains or when no drug was administered. Time to adequate antibiotics is defined as the time delay between a blood culture that became positive and the time adequate antibiotics were administered. In our ICU a restricted antibiotic strategy is conscientious followed [11]. The empiric antibiotic regimen administered is based on the underlying pathology, patients' history, local ecology, length of hospitalization, colonization status, presumed inciting focus, and hemodynamic status of the patient. Last-line antibiotics such as carbapenems and glycopeptides are only given to those patients colonized with multi-drug-resistant pathogens or those with a fulminated septic shock. Through mutual deliberation, different specialists (i.e. intensivist, infectious disease specialist, and microbiologist) daily verify whether narrowing the antibiotic spectrum is possible. Further, prophylactic antibiotics are only given preoperatively. For those already receiving antibiotic therapy, onset of BSI should be interpreted as a new outbreak of infection which on its turn will provoke an inflammatory response expressed by CRP and/or WCC changes in the blood. The source of bacteremia was determined by the attending intensivists and microbiologists, based on the presumed portal of entry of the isolated microorganism and by the clinical course. Blood sampling for chemistry is routinely collected each morning at 6 am. When multiple determinations were performed during a day, the highest level of CRP and/or WCC was taken into account.

Severity of illness was assessed by means of the Acute Physiology and Chronic Health Evaluation (APACHE) II score and determined daily as a surrogate marker of organ dysfunction aiming to evaluate a patients' clinical evolution. Haemodynamic instability was defined as dependence of vasoactive or inotropic therapy, acute renal failure as the need for haemodialysis, and respiratory failure as ventilation dependency during ICU stay [12-14].

### Statistical analysis

Variables are described as mean ± standard deviation (SD) and median (interquartile range). The Mann-Whitney *U *test and the Chi-square test were used as appropriate. A multivariable logistic regression was performed, including all variables having a *P*-value <0.10 in univariate analysis or with plausible relationship with both biomarkers to assess the impact of these determinants on the dynamics of CRP and WCC levels, respectively. Goodness-of-fit was tested by means of the Hosmer-Lemeshow statistic technique. Significance was accepted for a two-tailed *P*-value <0.05. For analysis, SPSS 12.0 (SPSS Inc., Chicago, IL, USA) software package was used.

The present study was approved by the Ethics Committee of the Ghent University Hospital (registration number: B67020072006).

## Results

**Figure 1:**
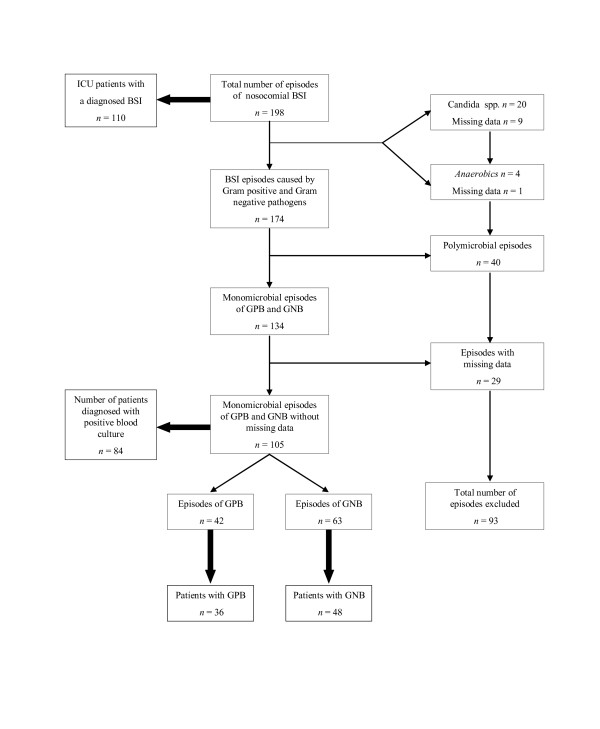
**Flow chart of exclusion criteria of episodes of nosocomial bacteremia (2003–2004)**. ICU, intensive care unit; BSI, bloodstream infection; GPB, Gram positive bacteremia; GNB, Gram negative bacteremia.

During the study period we recorded a total of 198 episodes of nosocomial BSI. Ninety-three episodes were excluded because of missing laboratory variables (n = 29), when polymicrobial (n = 40), or when fungal (n = 20) or anaerobic pathogens involved (n = 4). For 105 episodes (occurring in 84 patients) all data were available. Of these episodes, 42 were classified as Gram positive bacteremia (GPB) (43%) (occurring in 36 patients) and 63 as Gram negative bacteremia (GNB) (57%) (occurring in 48 patients) (Figure 1). Bacterial strains resistant to first line antibiotics were more prevalent encountered in GPB (*P *< 0.001).

Patients with GPB and patients with GNB had comparable age and sex distribution, organ failure during ICU stay, and severity of illness on ICU admission (Table 1). Overall, 90.5% vs. 90.5% of episodes were treated adequately (*P *= 0.999), however, there was a significant delay to initiation of adequate therapy in patients with Gram positive aetiology of bacteremia; 52.4% in patients with GPB compared to 76.6% in patients with GNB within 24-hrs after onset of bacteremia (*P *= 0.010), and 70.7% compared to 89.6% within 48-hrs (*P *= 0.037). Similarly, mean time delay to adequate therapy was 1.6 ± 1.6 days vs. 0.6 ± 0.8 days (*P *< 0.001) in GPB and GNB, respectively. Microbiological causes of BSI episodes are listed in Table 2.

**Table 1:** Patient baseline characteristics. SD, standard deviation; APACHE, Acute Physiology and Chronic Health Evaluation; ICU, intensive care unit; LOS, length of stay

**Characteristic**	**Episodes of Gram-positive bacteremia (*n *= 42)**	**Episodes of Gram-negative bacteremia (*n *= 63)**	***P***
Age, years, mean (SD)	55.6 ± 19.5	58.3 ± 15.2	0.905
Female, No (%)	15 (41.7)	16 (33.3)	0.497
APACHE II, mean (SD)	19.1 ± 6.7	20.5 ± 7.8	0.400
Comorbidity			
Liver disease, No (%)	1 (2.8)	0 (0.0)	0.429
Antibiotic therapy			
Adequate therapy ≤24-hrs, No (%)	22 (52.4)	48 (76.6)	0.010
Adequate therapy ≤48-hrs, No (%)	30 (70.7)	56 (89.6)	0.037
Adequate therapy, No (%)	38 (90.5)	57 (90.5)	0.999
Delay in the start of adequate therapy, mean (SD)	1.6 ± 1.6	0.6 ± 0.8	<0.001
Outcome			
ICU LOS, median (range)	16.5 (5.5–31.0)	19.5 (3.0–30.3)	0.726
ICU LOS before onset of bacteremia, median (range)	9.0 (1.3–24.0)	8.0 (2.3–17.8)	0.544
In-hospital mortality, No (%)	13 (36.1)	22 (45.8)	0.503

**Table 2:** Microbiological causes of nosocomial Gram positive and Gram negative bacteremia

**Organism**	***n***	**Number of antibiotic susceptible bacteria *n*(%)**
Gram-positive bacteria	42	9 (21.4)
*Staphylococcus *spp.	34	
*Staphylococcus aureus*	11	1 (2.4)
Coagulase-negative staphylococci	23	0 (0.0)
Streptococci/Enterococci	8	8 (19.0)
Gram-negative bacteria	63	53 (84.1)
*Enterobacter *spp.	20	13 (20.6)
*Pseudomonas aeruginosa*	16	15 (23.8)
*Klebsiella *spp.	8	8 (12.7)
*Escherichia coli*	6	6 (9.5)
*Proteus *spp.	5	5 (7.9)
*Serratia marcescens*	3	3 (4.8)
*Acitenobacter*	2	2 (3.2)
*Stenotrophomonas maltophilia*	1	0 (0.0)
*Morganella morganii*	1	1 (1.6)
*Flavo bacterium*	1	0 (0.0)

**Figure 2:**
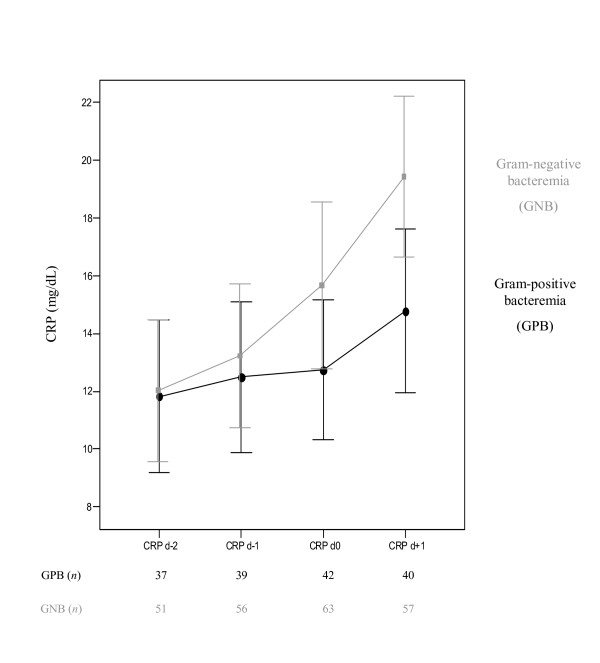
Dynamics of C-reactive protein serum concentrations in ICU patients with nosocomial bacteremia involving either Gram positive vs. Gram negative bacteria.

**Figure 3:**
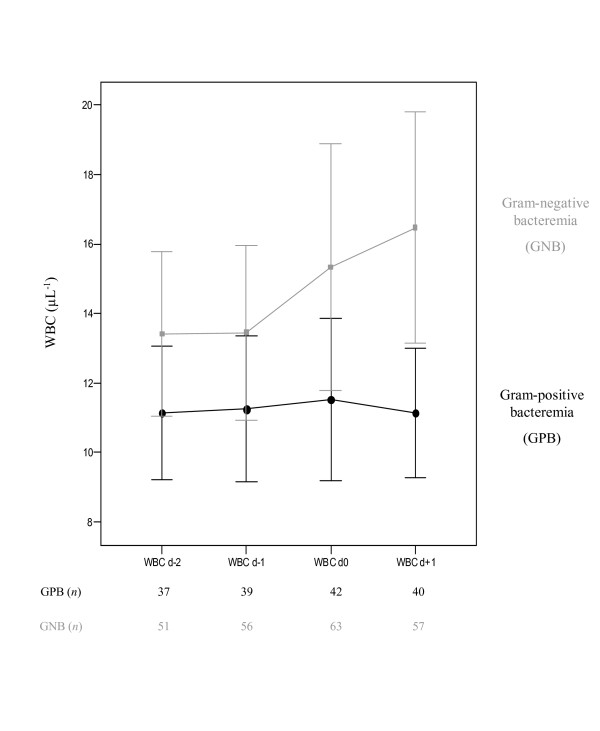
Dynamics of white blood cell count serum concentrations in ICU patients with nosocomial bacteremia involving either Gram positive vs. Gram negative bacteria.

Serum CRP concentrations were higher in patients with GNB than with GPB from d_0 _on. Moreover, in GNB patients these levels showed a steady and significant increase from d_-2 _onward with a peak concentration at d_1 _(*P *= 0.009), whereas in patients with GPB, these levels showed only a smooth increase (Figure 2). Evaluation of serial WCC levels showed comparable results (Figure 3). Analysis of the time course, as expressed by Δ CRP_d-2 to d+1 _and Δ WCC_d-2 to d+1 _levels, showed that median Δ CRP_d-2 to d+1 _levels for GPB and GNB were 3.1 mg/dL (-2.4 mg/dL-7.8 mg/dL) vs. 6.2 mg/dL (0.9 mg/dL-14.2 mg/dL) (*P *= 0.025), and median Δ WCC_d-2 to d+1 _levels were 180 × 10^3 ^cells/mm^3 ^(-2,100 × 10^3 ^cells/mm^3^-3,500 × 10^3 ^cells/mm^3^) vs. 1,900 × 10^3 ^cells/mm^3 ^(-1,500 × 10^3 ^cells/mm^3^-7,100 × 10^3 ^cells/mm^3^) (*P *= 0.171). We also verified whether an increase of CRP levels from d_-2 _to d_+1 _of 5 mg/dL, and whether an increase of WCC levels from d_-2 _to d_+1 _of 5,000 × 10^3 ^cells/mm^3 ^could differentiate between GPB and GNB. Both cutt-offs were exceeded in 9/36 (25.0%) and 8/36 (22.2%) of patients with GPB, compared to 35/48 (72.9%) and 36/48 (75.0%) in patients with GNB (*P *= 0.011 and *P *= 0.035, respectively).

Multivariate logistic regression analysis identified GNB (OR,5.06; 95% CI,1.52–16.91; *P *= 0.008) to be independently associated with a CRP level increase of 5 mg/dL from d_-2 _to d_+1_, whereas APACHE II score was not (OR,0.87; 95% CI,0.79–0.96; *P *= 0.006) (Goodness-of-fit; chi-square,2.46; df,8; *P *= 0.963). Gram negative aetiology of bacteremia (OR,3.31; 95% CI,0.96–11.41; *P *= 0.040) and APACHE II score (OR,1.10; 95% CI,1.00–1.20; *P *= 0.044) (Goodness-of-fit; chi-square,6.45; df,8; *P *= 0.587) were both independently associated with an increase of WCC levels from d_-2 _to d_+1 _of 5,000 × 10^3 ^cells/mm^3^. Focuses of infection as well as appropriateness of antibiotics were not shown to be independent determinants in the dynamics of both biomarkers (all *P *> 0.05).

## Discussion

Early initiation of adequate antimicrobial therapy is critical to ensure good outcome in severe infection [15-19]. Therefore, early recognition and accurate diagnosis of bacteremia is highly desirable, and appropriate antibiotics should not be delayed until blood cultures identify the offending pathogen [20]. However, increasing antimicrobial resistance and concerns about antibiotic exposure driving selection of multidrug-resistant strains have complicated both empiric antibiotic choices and clinical decisions whether to start antibiotics. The present study investigated whether Gram positive vs. Gram negative pathogens have different patterns in CRP and WCC count levels in order to help physicians in their daily clinical judgment.

Previous studies comparing groups of patients who received antibiotics because of documented vs. suspected infection, already evaluated serum concentrations of CRP and/or WCC counts in the diagnosis of infection [6,21-23]. However, most of these studies used the American College of Chest Physicians/Society of Critical Care Medicine Consensus Conference guidelines to define the presence or absence of sepsis [3,24]. Because these criteria are consensually considered to have high sensitivity and low specificity, we therefore selected a very homogeneous group of patients with unequivocal bacterial sepsis.

Although our cohort already had high baseline CRP and WCC count serum concentrations, some differential observation could be made after the onset of bacteremia with respect to both biomarkers. Patients with GPB demonstrated lower CRP and WCC levels at onset of bacteremia, which remained almost stable during the four study days. On the other hand, CRP and WCC levels at d_+1_, as well as the absolute increase between d_-2 _and d_+1_were significantly higher in patients with GNB. In both groups (patients with GPB vs. GNB) peak concentrations of CRP and WCC levels were observed one day following onset of bacteremia (*P *= 0.009, and *P *= 0.008), respectively. In accordance to earlier reports, by combining CRP and WCC measurements we could not further improve specificity for the diagnosis of bacteremia [6,25]. Using Δ CRP and Δ WCC levels in order to evaluate their respective evolution over time, multivariate analysis showed that Gram negative aetiology of bacteremia was independently associated with (i) an increase of CRP levels of 5 mg/dL, and (ii) an increase of WCC of 5,000 × 10^3 ^cells/mm^3 ^from two days prior until one day after onset of bacteremia, respectively. Remarkably, APACHE II score was, however, not independently associated with the first scenario (CRP increase), whereas it was independently associated with the latter (WCC increase). In both models, appropriateness of antibiotic therapy as well as focus of infection had no association with neither CRP nor with WCC.

Attributable mortality rates up to 45% have been reported in case of an inadequate choice of empirical therapy in patients with nosocomial bacteremia [26]. Because microbiological results generally only are available 48-hrs after blood cultures were obtained, and adjustment of inadequate therapy at this time poorly influences outcome, there is need for clinical or biochemical parameters to warn the clinician of an inadequate empiric choice at an earlier stage in the course [15,27]. In our study cohort, episodes of nosocomial BSI due to Gram positive bacteria received similar rates of adequate antibiotics compared with episodes of GNB; however, a substantial delay in the start was observed (*P *< 0.001). We noted a high rate (47.6%) of inadequate therapy within the first 24-hrs following onset of infection among episodes of GPB. This figure illustrates that particularly in this subgroup; considerable benefit could be achieved with respect to this time delay. Taken the relationship between the delay in starting adequate antimicrobial therapy and clinical outcome, a role for serial measurements of WCC and especially CRP serum concentrations in the early detection, as well as for follow up of bacteremia among critically ill patients may be presumed. Because in patients with GPB the highest rates of inadequate treatment are recorded, an increase of both CRP and WCC levels could be expected. However, this was not confirmed in our data. No significant differences were found in CRP and WCC levels according to the appropriateness of empirical antibiotic treatment. Although an adequate empirical antibiotic regimen is initiated, an increase of CRP and/or WCC levels is frequently observed over the next 24-hrs or even 48-hrs. Additionally, other events such as recent surgery, inflammatory insult, pancreatitis and trauma may cause fluctuations in CRP levels, respectively. Further, in the GPB-group, coagulase-negative Staphylococci were the most frequently isolated pathogens. In addition to the above, this may also explain the high observed rate of inadequate choice of empirical antibiotics. Though, since these pathogens are considered to be less virulent compared to other Gram positive strains such as *Staphylococcus *spp., serial measurements of CRP and WCC seem to be less relevant for this purpose.

The present study only investigated the dynamics of two variables (CRP and WCC) in their ability to differentiate between bacteremia caused by Gram positive vs. Gram negative pathogens. The short period of observation (4 days, respectively) includes another limitation of this study. Taken into account a longer period could have resulted in additional information (e.g. the amplitude of CRP and/or WCC levels could have been observed to be more pronounced before onset of bacteremia), though in an ICU setting, many other factors associated with increased CRP and/or WCC levels, however, not of infectious origin, are frequently observed. We used the APACHE II score as a surrogate marker of organ dysfunction; however, other scores aimed to assess severity of illness could have resulted in other conclusions. Also, we could not provide data with regard to previous antibiotic therapy in this cohort which might have biased our results. Because our findings are based on a single centre study, one should be careful when interpreting or extrapolating these data. Next, other physical or laboratory criteria such as fever, hypothermia, tachycardia, tachypnea, inflammatory mediators (e.g. interleukine-6, tumor necrosis factor-α) were not evaluated and might be more straightforward in this assessment. Lastly, as this study was conducted retrospectively, it is recommended to repeat this study with a prospective design. Nonetheless, this study is the first to elaborate on the dynamics of CRP and WCC levels in adult ICU patients with GPB and GNB.

## Conclusion

In conclusion, surveillance of bacteremia in the ICU is crucial in detecting major changes in aetiology such as the increasing incidence of GPB. Our findings suggest that obvious different patterns are observed in patients with nosocomial GPB vs. GNB, with respect to both biomarkers studied. However, their use is less relevant in guiding antibiotic therapy.

## Competing interests

The author(s) declare that they have no competing interests.

## Authors' contributions

DMV collected and analyzed the data, developed the study design and was principal writer of the manuscript. EAH participated in developing our main research questions and contributed in statistical analyses. POD and SIB were involved in critical analysis and writing the manuscript. SIB also collaborated with extracting necessary data. RAP and JDC critically reviewed the manuscript and were involved because of their extraordinary expertise in the field. All authors read and approved the final version of the manuscript.

## Pre-publication history

The pre-publication history for this paper can be accessed here:


